# Exercise performed around MLSS decreases systolic blood pressure and increases aerobic fitness in hypertensive rats

**DOI:** 10.1186/s12899-015-0015-7

**Published:** 2015-03-14

**Authors:** Bernardo A Petriz, Jeeser A Almeida, Clarissa P C Gomes, Carlos Ernesto, Rinaldo W Pereira, Octavio L Franco

**Affiliations:** Centro de Análises Proteômicas e Bioquímicas, Programa de Pós-Graduação em Ciências Genômicas e Biotecnologia, Universidade Católica de Brasília SGAN, Quadra 916, Módulo B, Av. W5 Norte, CEP 70.790-160 Brasília, DF Brasil; Laboratório de Avaliação Física e Treinamento – LAFIT, Universidade Católica de Brasília, Brasília, DF Brasil; UDF – Centro Universitário de Brasília, Brasília, DF Brasil; S-Inova, Pos-Graduação em Biotecnologia, Universidade Católica Dom Bosco, Campo Grande, MS Brasil; Departamento de Educação Física, Universidade Federal de Mato Grosso do Sul, Campo Grande, MS Brasil

**Keywords:** Blood pressure, Hypertension, SHR, Maximal lactate steady state, Treadmill running exercise, Incremental test, Vmax

## Abstract

**Background:**

Exercise is a non-pharmacologic agent widely used for hypertension control, where low intensity is often associated with blood pressure reduction. Maximal lactate steady state (MLSS) was recently identified in spontaneously hypertensive rats (SHRs) as an important step in establishing secure intensities for prescribing exercise for hypertensive phenotypes. Here we verified the effects of training around MLSS, 20% below MLSS, and 15% above MLSS on aerobic fitness and blood pressure status of SHR. Eighteen-week-old SHRs (n = 5, ~ 172.4 ± 8.1 mm Hg systolic blood pressure) were trained on a treadmill for 4 weeks for 30 min/day, 5 days/week at a velocity of 20 m.min^−1^. After training, a novel MLSS and incremental test was performed to evaluate the animals’ aerobic fitness. Furthermore, ~ 22-week-old SHRs (n = 12, ~169.8 ± 13.8 mm Hg systolic blood pressure) were divided into non-exercised (CG, n = 4), low intensity (LIG, n = 4) and high intensity (HIG, n = 4) groups, where rats were trained at 16 m.min^−1^ and 23 m.min^−1^ respectively for 30 min/day, 5 days/week for 4 weeks.

**Results:**

Exercise performed at MLSS enhanced aerobic fitness, leading to a novel MLSS, identified around 30 m.min^−1^. Low and high intensity training reduced systolic blood pressure and only high intensity training led to improved aerobic fitness (28.1%, p < 0.01).

**Conclusions:**

Therefore, our data indicate a decrease in blood pressure due to low and high exercise intensity, and an increase in aerobic fitness provided by high-intensity exercise in SHRs.

## Background

Hypertension is a multifactor disease that has a high epidemiological correlation with cardiovascular disease and other pathologies [1,2], being a major public health concern [3]. Systematic exercise stimulus is shown to be a pharmacologic-independent treatment of hypertension, since the effect of exercise can reduce high blood pressure rates and cardiovascular mortality [4]. Endurance training promotes endothelial vasodilatation [5] and induces eccentric physiologic heart hypertrophy, which delays and attenuates pathologic heart hypertrophy [6,7] and systolic dysfunction in hypertensive phenotype rodents [8].

The nature and intensity of exercise are critical points in the magnitude of physiological effects on the cardiovascular system, such as improvement in aerobic fitness. However, the response of blood pressure (BP) in hypertensive phenotypes to post-exercise effects remains a contradictory topic: a large set of physiological responses has been reported due to the variety of exercise types and protocols reviewed by [9]. On the one hand, it has been reported that exercise did not lower blood pressure [9], but on the other, contradictory data have shown a systolic lowering effect [10]. However, the variety of protocols to test these parameters must be considered. Thus, the response of blood pressure to different exercise magnitudes remains a contradictory topic.

Nevertheless, the reduction in BP may be exercise intensity-dependent; and different exercise intensities seem to be a major aspect in the control and adequate treatment of hypertension. Recently, our group has identified the maximal lactate steady state (MLSS) – a gold standard methodology to assess aerobic fitness [11] – in spontaneously hypertensive rats (SHR) [12]. Considering that, adequate exercise prescription and controlled exercise intensity are of prime importance for hypertension treatment; here we investigated the effect of exercise training at MLSS, below and above MLSS intensity on aerobic fitness (Vmax) and BP in SHRs.

## Methods

### Animals and initial procedures

The present study was divided into two distinct experiments. The first was composed of 5 female spontaneously hypertensive rats (SHRs) of ~18 weeks old. The second experiment was composed of 12 male SHRs of ~22 weeks old divided into three experimental groups: control group (CG; n = 4), exercised at low intensity (LIG; n = 4), and exercised at high intensity (HIG; n = 4). All animals were obtained from the bioterium of the Federal University of São Paulo, Brazil. Water and food were provided *ad libitum* and the animals were kept in a 12:12 h dark–light cycle in a room at 23 ± 2°C. The study was approved by the local ethics committee on animal use from the Catholic University of Brasília, Brazil, and procedures were in accordance with the Brazilian College of Animal Experimentation [13]. Before beginning the exercise training, all animals were familiarized with the experimental environment and treadmill platform (Li 870, Letica Scientific Instruments, Barcelona, Spain) and adapted for three weeks as previously described [12].

### Experimental design 1

After the adaptation period, five rats (~18 weeks old, 227.4 ± 29.3 g, and 172.4 ± 8.1 mmHg of systolic blood pressure) were submitted to 4 weeks of treadmill running, 5 days per week, 30 min per day at a velocity corresponding to the MLSS (20 m.min^−1^).

### Maximal lactate steady state

**Figure 1:**
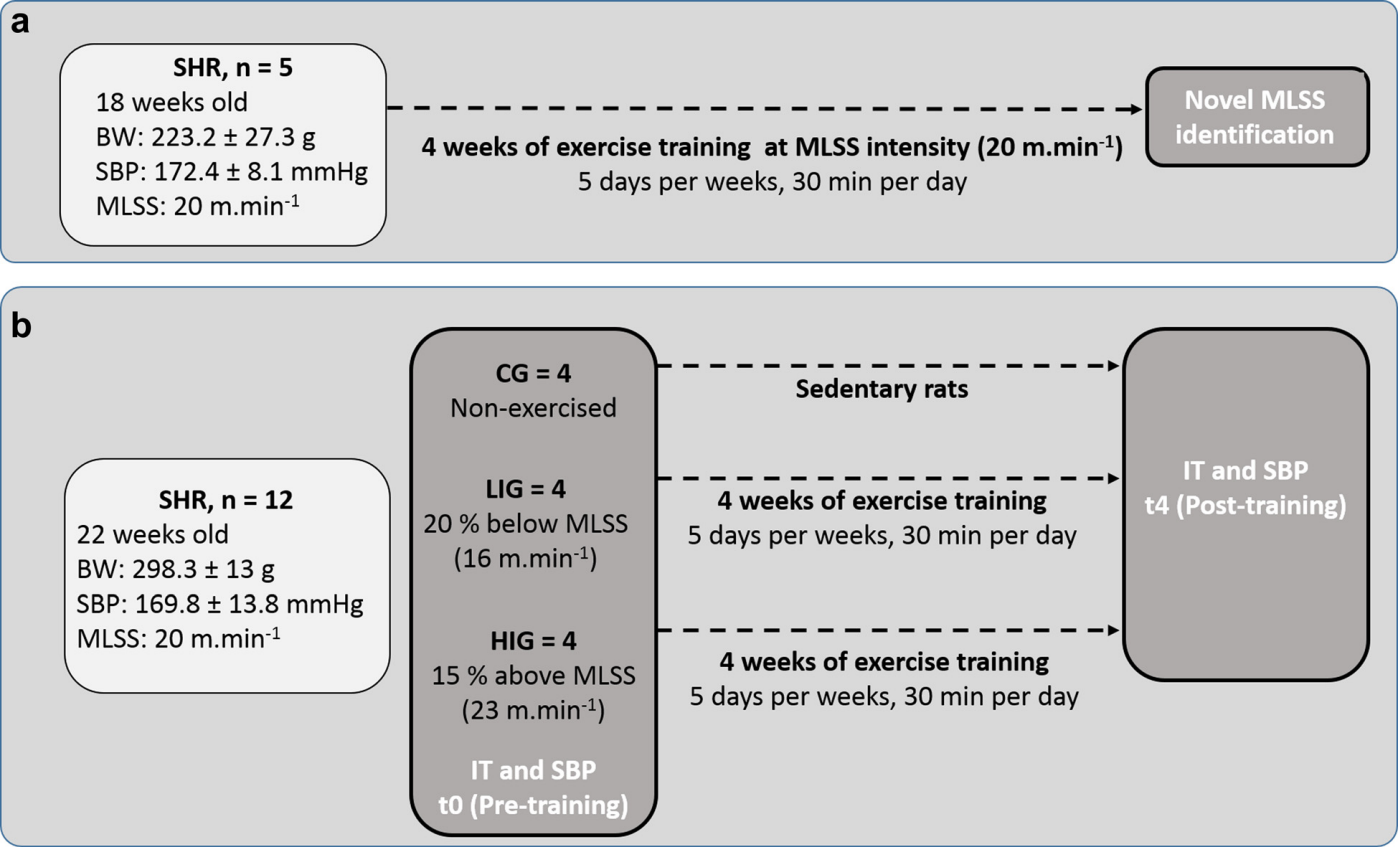
**Experimental design.** Experimental design of the two experiments conducted in the present study. The first experiment **(a)** aimed to verify the effectiveness of four weeks of exercise training performed at a relative intensity at MLSS (20 m.min^−1^) previously identified in SHRs. The second experiment **(b)** aimed to verify the effect of exercise training at two distinct exercise intensities (low and high intensity) on aerobic fitness and blood pressure of SHRs. The effect of exercise intensities on aerobic fitness and systolic blood pressure (SBP) was assessed by comparing incremental tests of maximal velocity (incremental tests) and SBP in pre (t0) and post training period (t4).

The MLSS was previously identified by Almeida, et al. [12] during rectangular tests at three different velocities (15 m.min^−1^, 20 m.min^−1^ and 25 m.min^−1^). In order to verify the effects of the proposed exercise intensity on MLSS at the end of the training period (4 weeks) a novel MLSS was tested in all animals using three other velocities (25 m.min^−1^, 30 m.min^−1^ and 35 m.min^−1^) (Figure 1a). The velocities were set randomly and the tests were carried out at 48 h intervals. The tests lasted for 25 min of continuous exercise (0% graded) or until animal exhaustion. During the tests, capillary blood was collected every 5 min from the distal portion of the animals’ tail for blood lactate concentration analysis. MLSS was considered as the highest intensity of effort where the blood lactate did not vary more than 1 mmol.L^−1^ from the 10th to the 25th min, as described previously [12].

### Blood lactate analysis

10 μL of blood was collected with capillaries from a small incision in the distal tail portion of each animal, rapidly deposited in microtubes (0.6 mL) containing 20 μL of 1% sodium fluoride, and stored at −20°C for further biochemical analysis. The blood lactate concentration was analysed by the electro-enzymatic method from YSI Sports 2700 (Yellow Springs, OH, USA) [14].

### Experimental design 2

At the end of the first experiment, a novel group of 12 SHRs (~22 weeks old, 306.8 ± 11.1 g, and 169.8 ± 13.8 mm Hg of systolic blood pressure) was used to verify the effect of two distinct exercise intensities (below and above MLSS) on aerobic fitness and systolic blood pressure status. SHRs were divided into three groups: low intensity group (LIG; n = 4), which trained at a running velocity corresponding to 20% below MLSS (16 m.min^−1^), high intensity group (HIG; n = 4), which trained at a velocity corresponding to 15% above the MLSS (23 m.min^−1^), and control group (CG; n = 4), where the animals were not submitted to exercise. The exercise groups underwent 30 min of treadmill exercise (without slope), 5 days per week for 4 weeks.

### Incremental test (IT)

Besides the use of MLSS as parameter for exercise prescription, an incremental test (IT) (0% graded test, increments of 3 m.min^−1^ every 3 min, starting at 5 m.min^−1^ until animal exhaustion) was also used to determine the maximum velocity (Vmax) in all groups (CG, LIG and HIG). IT was performed previously to the training period (t0) and immediately after four weeks of exercise training (t4) (Figure 1b). In this way, Vmax was used to establish aerobic fitness.

### Blood pressure measurements

Systolic blood pressure (SBP) was measured in all animals before the training period started (t0) and at the end of four weeks of exercise training (t4). To carry out the SBP measurements, all animals were lightly soothed with a common combination of 10% ketamine (10 mg.kg^−1^) and 2% xylazine (10 mg.kg^−1^), and then SBP was measured by the tail-cuff plethysmography method (LE 5001 Pressure Meter, Letica, Barcelona, Spain). Inconsistencies in diastolic blood pressure were observed throughout the experiment, but these data were not recorded in this study.

### Statistical procedures

After verifying data normality (Kolmogorov-Smirnov test), data were presented as mean and standard deviation values in both experiments. Here, the parametric test was able to identify the data normality in a sample size of 4. To observe the effect of exercise training at MLSS intensity on aerobic fitness and the effect of different exercise intensities on SBP, inferential analyses were conducted by One-way ANOVA with Bonferroni *post-hoc* test. The level of significance was set at P < 0.05.

## Results

### Effect of treadmill training at MLSS intensity

In relation to the effectiveness of four weeks of exercise training at a relative intensity at MLSS (20 m.min^−1^), MLSS was once again identified after the training period (Figure 1a). Here it was shown that MLSS velocity enhanced from 20 m.min^−1^ to 30 m.min^−1^ with 3.8 ± 0.3 mmol.L^−1^ of [Lac]. On the other hand, the running velocity used above this intensity (35 m.min-1) did not show the stabilization of [Lac], which demonstrated changes up to 1 mmol.L^−1^ during the exercise period. Compared to the previous MLSS identification (20 m.min^−1^), all rats showed an increase in aerobic fitness by ~ 50%.

### Effect of treadmill training below and above MLSS intensity

Regarding the second group of hypertensive rats (LIG and HIG), an incremental test (IT) was also applied before the training period (t0) and at the end of the four weeks of training (t4). According to Table 1, IT (t0) indicated no differences in aerobic fitness between the groups (CG, LIG, and HIG, P > 0.05). Only the group of animals that trained at high intensity (15% above MLSS) – corresponding to 80% of the maximal velocity (Vmax) – showed a significant increase in aerobic fitness (t0; 26 ± 4.2 vs. t4; 33.3 ± 1.7 m.min^−1^, P < 0.05) compared to CG and LIG.

**Table 1:** **Maximal Velocity during Incremental test (IT) at moments of exercise training pre (t0) and post-4 weeks (t4)**

**Group**	**IT pre-training (t0) (m.min** ^**−1**^ **)**	**IT post-training (t4) (m.min** ^**−1**^ **)**	Δ**%**	***p-value***
**CG (** ***n*** **= 4)**	26.5 ± 2.2	25.5 ± 2.5	−3.8	0.5
**LIG (** ***n*** **= 4)**	27.2 ± 2.7	29.1 ± 2.1	7.0	0.26
**HIG (** ***n*** **= 4)**	26 ± 4.2	33.3 ± 1.7*	28.1	0.01

Legend: CG; control group, LIG; Low Intensity Group, HIG; High Intensity Group, IT; maximal velocity incremental test, *velocity higher compare to IT pre-training (t0) p < 0.05.

### Effect of treadmill training below and above MLSS intensity on SBP

**Figure 2:**
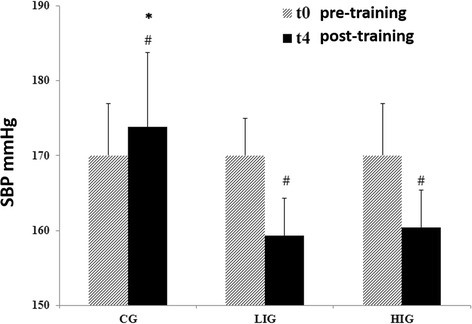
**Effect of exercise training on systolic blood pressure.** Systolic blood pressure (SBP) was measured in all animals previous to the training period (t0) and at the end of four weeks of exercise training (t4). Rats started the experiment with SBP above 160 mm Hg, indicating the prevalence of high blood pressure. Both exercise intensities reduced SBP significantly (LIG and HIG). The differences in SBP pre (t0) and post training (t4) are indicated with (#) symbol (P < 0.05), and (*) indicates a significant difference in SBP after the exercise period (t0) between CG and trained groups (LIG and HIG) (P < 0.05).

SBP from all hypertensive rats subjected to exercise training (below and above MLSS) significantly decreased when compared to the non-exercised CG (P < 0.05). In addition, at t4, the LIG and HIG showed significant reductions in SBP in relation to t0 (~9.6 and ~10.7 mm Hg, respectively). On the other hand, the CG SBP increased ~4 mm Hg (P < 0.05) (Figure 2), confirming the attenuating effect of exercise on SBP during the aging process in SHR (Figure 2).

## Discussion

SHR is a well-known animal model and widely used in exercise research [6]. A wide range of protocols involving different exercise durations and intensities have been used to assess the antihypertensive effects of exercise, as well as the attenuation of hypertensive cardiac hypertrophy [9,10,15]. Regardless of the known effect of exercise on high blood pressure reduction, it seems that this effect could be dependent on the magnitude of exercise stimulus; larger reductions in BP are observed in response to lower exercise intensities [10,16-18]. However, studies with hypertensive rodents (SHR) suggest that other factors, such as age and hypertension stage, may impair the lowering effect of exercise on blood pressure, which can appear to be unaffected, as described in the meta-analysis by Schluter et al. [9]. It was also observed that the blood pressure of normotensive rodents responds less significantly to aerobic training compared to hypertensive phenotypes such as SHRs [10].

Although several studies have shown a reduction in blood pressure through exercise stimulus [10,15], few have investigated this response at different exercise intensities [18]. Therefore, this is the first study to use controlled exercise intensities based on MLSS to assess its effect on aerobic fitness and blood pressure in hypertensive rats.

**Figure 3:**
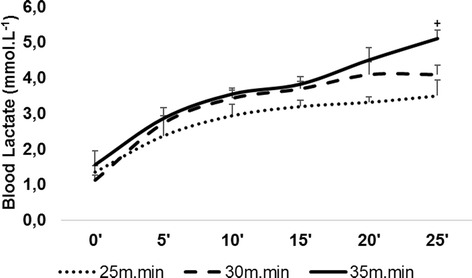
**MLSS determination after 4 weeks of exercise training.** MLSS identification was performed with three different running velocities (25 m.min^−1^, 30 m.min^−1^, and 35 m.min^−1^), where MLSS was identified at a velocity corresponding to 30 m.min^−1^ with 3.8 ± 0.3 mmol.L^−1^ of [Lac], where the running velocity above this intensity (35 m.min^−1^) did not show the stabilization of [Lac] up to 1 mmol.L^−1^ during the exercise period. Blood lactate concentration collected in each velocity test is presented in mean values with SD. (^+^) [Lac] more than 1 mmol.L^−1^ compared to 10th min.

MLSS determination is of prime importance since it is the gold standard in assessing aerobic fitness and is widely used for exercise prescription [11]. The MLSS was recently identified for SHRs [12] and obese Zucker rats [19], showing that this intensity improves aerobic fitness in the obese animals [20]. Here we also demonstrated that four weeks of exercise training at intensities around the MLSS was also effective to enhance aerobic fitness in these hypertensive rodents (Figure 3). Exercise intensity equivalent to the MLSS, which correlates closely with lactate threshold [21,22], has been prescribed as being of moderate intensity, often used in exercise programs for special groups (e.g. diabetes, obesity, and hypertension) [23,24]. Therefore, our data reinforce the importance of determining adequate exercise intensity and the efficiency of MLSS exercise-based intensities for hypertensive individuals.

The present study also demonstrated that exercise training at intensities below (LIG) and above (HIG) MLSS was able to reduce SBP in SHRs (Figure 2), while only high intensity exercise improved aerobic fitness (Figure 3). It is believed that intensities above the anaerobic threshold (e.g. 15% above the MLSS) are more effective in increasing aerobic fitness and leading to improvement in aerobic power compared with intensities below the MLSS. Recently, 6 weeks of training (20 min/day, 7 days/week) at low running intensity (30% of maximal aerobic velocity) but not moderate intensity (60%) was shown to significantly reduce blood pressure in male and female SHRs (10 months old) with severe hypertension [18]. In our study, a lower intensity exercise (9–10 m.min^−1^ vs. 18 m.min^−1^) was able to significantly reduce severe high blood pressure (p < 0.05). Other authors have also established moderate intensity at 60% of maximal aerobic velocity – corresponding to 18–20 m.min^−1^ –, which is around the identified MLSS in SHRs and Wistar rats [12]. Although we showed a decrease in blood pressure after high-intensity exercise training (4 weeks, 5 days/week, 30 min/day at 23 m.min^−1^) (Figure 2), the moderate exercise intensity used by Sun, et al. [18] did not lead to blood pressure reduction, even after prolonged exercise (6 weeks vs. 4 weeks).

A high exercise intensity (60 min, 5 sessions per week, for 12 weeks, at running speed gradually increased by 3 m.min^−1^ until 27 m.min^−1^) proposed in the study of Huang, et al. [15] also reduced SBP in SHRs. Citrate synthase activity was significantly enhanced in trained animals, indicating an improvement in aerobic fitness. However, in this mentioned study, animals trained at a greater volume (60 min/session for 12 weeks) and at a higher running velocity (27 m.min^−1^), which corresponds to 35% above the MLSS previously identified for SHRs [12]. Thus, these animals may have trained in a severe intensity domain, indicating that such intensity reduces blood pressure. A single session of high-intensity running exercise (30 m.min^−1^ until exhaustion) was also shown to induce vasorelaxant responses in trained SHRs [5], indicating the acute effect of exercise intensities above MLSS. Melo, et al. [10] showed that 13 weeks of running exercise at low intensity (50–60% of maximal exercise capacity) reduced blood pressure in SHRs compared to non-trained SHRs (176 ± 1 vs. 190 ± 1 mm Hg, P < 0.05) and increased exercise performance. It was also indicated that the proposed low exercise intensity was effective in normalizing arteriole wall/lumen ratio in skeletal muscles, led to thinner myocardium arterioles, and increased capillary profile in these animals. The authors suggested that these compensatory adjustments might have contributed to the reduction in blood pressure by reducing local resistance and improving muscle circulation [10].

Research using different exercise intensities supports the concept of vasodilation dependent on exercise intensity. Although low exercise intensity is often more associated with the attenuation of high blood pressure, higher intensities (above MLSS) are also shown to play an antihypertensive effect, as suggested by our results and those of others [15]. However, research involving experimental animals limits the idea of higher exercise intensities as a therapeutic factor for chronic hypertension control. Indeed, da Costa Rebelo, et al. [25] indicates that high-intensity aerobic exercise is associated with cardiac fibrosis and acceleration of hypertensive heart disease, which draws attention to higher intensities as a risk factor rather than a cardioprotective effect.

## Conclusion

One limitation of this study is the low number of animal replicates, although the sample size and its normality were evaluated by a parametric test. Moreover, it is also believed that inbred rats may significantly influence the similarity of their characteristic, and this may be a feature of our study. However, the present study confirms the previously identified MLSS as an adequate intensity to improve aerobic fitness in SHRs and demonstrates that four weeks of aerobic exercise performed 20% below MLSS was able to reduce blood pressure independent of improving aerobic fitness. Our data support the therapeutic potential of low exercise intensity based on MLSS in lowering blood pressure in hypertensive phenotypes. Further analyses must consider training at MLSS intensity and further investigate the role of higher intensities in blood pressure and cardiac remodeling in hypertensive phenotypes. Besides the intensity factor, exercise volume and voluntary exercise are also dynamics that must be considered in experimental design with animal models.
